# Location of water in fresh sugarcane bagasse observed by synchrotron X-ray microtomography

**DOI:** 10.1371/journal.pone.0208219

**Published:** 2018-12-06

**Authors:** Carlos E. Driemeier, Liu Y. Ling, Daison Yancy-Caballero, Paulo E. Mantelatto, Carlos S. B. Dias, Nathaly L. Archilha

**Affiliations:** 1 Brazilian Bioethanol Science and Technology Laboratory (CTBE), Brazilian Center for Research in Energy and Materials (CNPEM), Campinas, SP, Brazil; 2 Institute of Mathematics and Statistics (IME), University of São Paulo (USP), São Paulo, SP, Brazil; 3 Brazilian Synchrotron Light Laboratory (LNLS), Brazilian Center for Research in Energy and Materials (CNPEM), Campinas, SP, Brazil; College of Agricultural Sciences, UNITED STATES

## Abstract

Sugarcane bagasse is a vast lignocellulosic byproduct generated in the industry with ~50% humidity (1 kg dry matter associated with 1 kg water). Although the presence of water brings deleterious consequences for combustion, storage and sugar extraction, the location of water in fresh bagasse remains unknown. In this work, we use synchrotron X-ray microtomography for non-invasive 3D imaging of fresh bagasse particles, which allows the visualization of intraparticle water. The sclerified fiber cells in the sheaths surrounding xylem vessels are often found full of water. We suggest this can be juice preserved from the native stalks as many sclerified fibers seem to keep their structural integrity despite the mechanical action during sugarcane crushing. The microtomograms of fresh bagasse also shows mineral particles adhered to biomass surfaces, with adhesion presumably favored by the presence of water. In summary, this work unveils the location of water in fresh bagasse, solving an old mystery of sugarcane technology.

## Introduction

Sugarcane is produced in many tropical and subtropical countries around the world, with global annual production of 1.9 billion metric tons [1]. About 12–14% of this mass (250 million metric tons) corresponds to the lignocellulosic matter in the sugarcane stalks, which is transformed in bagasse after the stalks are crushed to extract the juice [2]. Considering the above numbers and a lower heating value of 17 MJ/kg for dry bagasse, this agricultural residue could hypothetically supply 4 EJ of energy per year. This would correspond to a share of 6% in the global supply of bioenergy of 63 EJ [3]. Although the numbers demonstrate the great potential of bagasse in both mass and energy basis, harnessing the full potential faces several limitations such as the inherent presence of residual water. In the sugarcane industry, bagasse comes out of the process with ~50% humidity (wet basis) [4,5], meaning that 1 kg dry matter is impregnated with about 1 kg water. Surprisingly considering the long tradition of the sugarcane technology, the location of water in fresh bagasse remains unknown.

The presence of water in bagasse has several important consequences. When fresh bagasse is used as boiler fuel, which is common practice in mills, water evaporation (ΔH = 2.3 MJ/kg) consumes about 13% of the calorific value and reduces the combustion temperature, impairing efficiencies [5]. Sugar extraction provides another interesting perspective. Industrial extraction efficiencies are about 94–98% [5] and the residual 2–6% of sugars are in some way related to the water remaining in bagasse. Concerning bagasse storage, mills often stockpile part of their fresh bagasse and the presence of water is critical for all sorts of biological degradation mechanisms taking place during storage [6]. Also, water in bagasse is important considering water utilization. Despite the environmental pressure to improve water use efficiency in the sugarcane industry, water in bagasse is wasted by evaporation, either at the stockpile or in the boiler.

**Fig 1:**
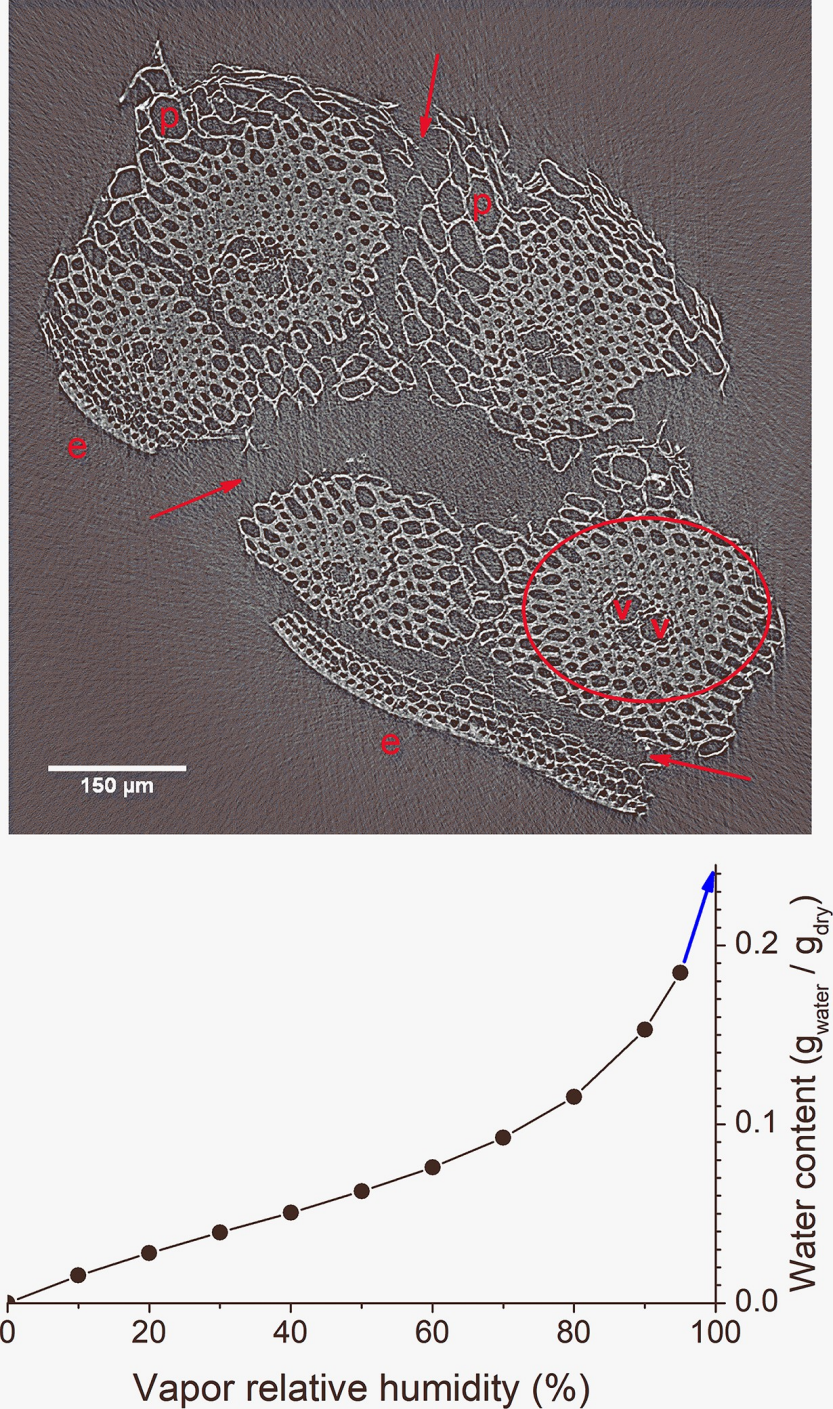
Distinct environments for location of water in bagasse. (Top) Cross-section image of a dry bagasse particle. Water can hydrate the cell walls (light gray) and fill intracellular voids (dark gray). Distinct tissues are identified: parenchyma cells (p), epidermis region (e) and vascular bundle (ellipse) containing a pair of xylem vessels (v). Tissue cracks are indicated (red arrows). (Bottom) Moisture sorption isotherm can be extrapolated (blue arrow) to indicate the approximate amount of water that saturates the cell walls.

Clues about the location of water in bagasse can be learned from the basics of biomass hydration. Water inside the biomass can be in two types of environments: (*i*) hydrating the cell walls and (*ii*) filling intracellular voids (*i*.*e*., cell *lumina*) [7]. The distinction between these two types of environments is readily seen in bagasse cross-section images (Fig 1) [8]. The amount of water associated with cell wall hydration can be estimated by extrapolation of moisture sorption isotherms (Fig 1, data from Ref. [9]), which indicates saturation at ~0.25 g_water_/g_dry_. This is similar to the so-called fiber saturation point observed in woods [7]. Converting to wet-basis percentage, this water content corresponds to ~20% humidity (100%×0.25/1.25). On the other hand, the amount of juice in fresh stalks (~87%) estimates the percentage of water that would be hypothetically observed in bagasse if all the cell walls and cell *lumina* were fully saturated. With that said, we must note the ≈50% humidity of fresh bagasse is in-between these limits: it is higher than cell wall saturation (~20%), but lower than full filling of all the cells (~87%). Therefore, we can anticipate fresh bagasse has only part of the cells filled with water.

In this work, we use synchrotron X-ray microtomography [8,10] to perform non-invasive imaging of the interior of fresh bagasse particles. We visualize the water volumes inside bagasse and confirm that cells are partly filled with water. We also identify which tissues are preferentially wet or dry. Moreover, we observe and analyze the mineral particles found in association with wet bagasse. The results provide novel insights about water-related phenomena taking place during sugarcane crushing and indicate new possibilities for valorization of this renewable bioresource.

## Materials and methods

### Collection of wet bagasse

On June 5^th^, 2017, 9:00 a.m., fresh sugarcane bagasse was collected at the industrial site of Usina Granelli (coordinates: -22.5189°, -47.7138°), Charqueadas, São Paulo, Brazil. The mill was operating normally. The mill analytical bulletin had recorded bagasse humidity between 46–53% during the previous 24 hours, demonstrating that the collected bagasse had the typical range of humidity. In this mill, crushing of sugarcane stalks is performed by a shredder followed by a milling tandem with five mills, each mill composed by three rolls. Warm bagasse was collected from the conveyor belt after the last mill. It was then inserted into a sealed thermalized bag containing bottled ice. This storage condition cooled down the bagasse and minimized any evaporative loss of water. The wet bagasse was immediately transported to the Brazilian Synchrotron Light Laboratory (LNLS/CNPEM) distant 106 km from Usina Granelli. After adjusting beamline conditions, 3D images of wet bagasse were successfully acquired from 11:45 a.m. to 20:00 p.m., that is, only a few hours after fresh bagasse was collected.

### Image acquisition and reconstruction

X-ray microtomograms were acquired in the IMX beamline of LNLS/CNPEM. Imaging conditions were adjusted for wet samples, having a previous study of dry bagasse as reference [8]. Wet fibrous particles were manipulated with tweezers and inserted into ø = 1.6 mm Kapton tubes, which were vertically aligned and attached to the sample holder. To avoid water loss during X-ray exposure, both sides of the tube were sealed, thus encapsulating the samples.

The transmission images of the bagasse were obtained using radiation from the 1.67 T bending magnet of the 1.37 GeV UVX storage ring. The setup produced a polychromatic beam with peak energy at approximately 8 keV and approximately 50% bandwidth. The projections were recorded by an indirect detector system, based on a 50 μm thick LuAg:Ce scintillator that converts the transmitted X-rays into visible light. The light is focused on a PCO2000 CCD sensor by an infinity corrected optics, which can produce an adjustable magnification of the visible light image radiating from the scintillator, yielding X-ray projection images with effective 1024×1024 pixels (2×2 binned) and equivalent pixel sizes of 1.64×1.64 μm^2^. This setup allowed for an exposure time of only 25 ms per projection image, thus making the whole measurement fast enough to prevent water evaporation.

**Fig 2:**
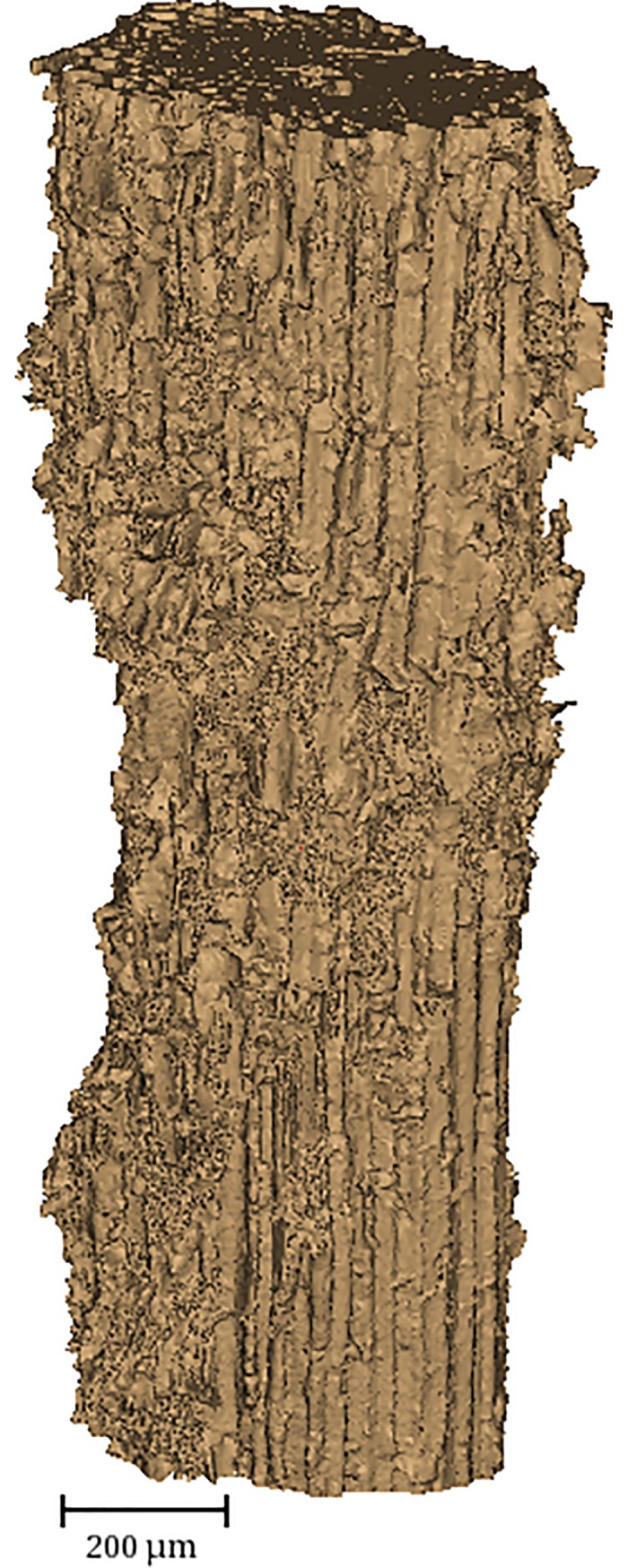
3D visualization of a selected bagasse particle.

Imaged segments were typically ~mm away from the longitudinal extremes of the particles. Each 3D reconstruction used 1001 equally spaced projection images acquired by sample rotation from 0° to 180°. 3D image reconstruction used the in-house developed Reconstruction Algorithms for Tomography (RAFT) [11]. A rendered visualization of a bagasse particle is shown in Fig 2 to exemplify the type of object captured by the microtomography analysis.

### Sampling and classification of bagasse particles

Bagasse is a heterogeneous particulate containing fines and fibrous particles, including coarse fibers with size of centimeters, which are much larger than the FOV of microtomography. Hence, the limited representativeness of the acquired images is an inherent issue in microtomographic studies of bagasse. As in the previous study [8], this limitation was approached in two ways. First, we selected a broad range of fibrous particles for 3D imaging, aiming at having all the major tissues represented, except the disaggregated parenchyma cells that form the non-fibrous fines. Second, we classified the 3D images in terms of three types of fibrous particles: *i) Rind* particles show vascular bundles (VBs) from sugarcane internode in association with epidermis regions; *ii) Pith* particles show VBs from sugarcane internode without visible association to epidermis; *iii) Undetermined* particles show other types of tissues, which may originate from sugarcane tops, leaves, or any other vegetal impurities. In addition to this classification, we counted the number of VBs appearing in the cross-sections of each particle, further informing about the characteristics of the particles selected for imaging.

### Analysis of mineral particles

Mineral particles are observed as high-contrast structures in microtomograms of sugarcane bagasse [8]. Segmentation and morphometry of the mineral particles used the Fiji [12] distribution of ImageJ [13]. Image preprocessing applied 3D Gaussian filter with sigma = 2.0. Segmentation was performed using Python scripts that call functions of the Morphological Segmentation plugin from the MorphoLibJ library [14], with the options for object image, morphological gradient of radius 1 and connectivity 6. The dynamic value (tolerance) was varied in preliminary trials followed by visual checks. Then, an optimal tolerance of 21 was chosen for the analysis. Segmented mineral particles touching the borders of the image or having low contrast (*i*.*e*., inappropriate segmentation) were excluded from the morphometric analysis. Morphometry of the mineral particles was performed with the MorphoLibJ [14]. The following metrics were calculated: volume, sphericity, and semi-axis lengths R_1_, R_2_ and R_3_ (major, intermediate, and minor axis, respectively) of the greatest ellipsoid that fits inside the mineral particle. Ellipsoid factor (EF) was calculated from semi-axis lengths (EF = R_3_/R_2_ – R_2_/R_1_), ranging from -1 for strongly disk-shaped ellipsoids, to +1 for strongly rod-shaped ellipsoids.

## Results and discussion

### Classification of bagasse particles

A total of 23 particles of fresh bagasse were successfully imaged, counting 8 particles classified as rind, 13 as pith, and 2 of undetermined tissue type. As expected, the rind particles are larger and, on average, have more VBs in their cross sections. The number of VBs visualized in rind particles range from 1 to 5, while in pith particles the number of VBs range from 1 to 3 and most pith particles (8 out of 13) show a single VB (Table 1). As mentioned (section 2.3), the imaged particles cannot be considered statistically representative of the whole bagasse. Nevertheless, the main types of fibrous particles and their typical features are represented in the image set and, therefore, they can provide the insights about water location in fresh sugarcane bagasse.

**Table 1:** Classification of the 23 imaged bagasse particles in terms of particle type and number of vascular bundles (VBs) visualized in the cross-sections.

Particle type	Number of VBs					
	1	2	3	4	5	Total
**Rind**	1	1	3	2	1	8
**Pith**	8	3	2	0	0	13
**Undetermined**	2	0	0	0	0	2

### Visualization of water in bagasse

**Fig 3:**
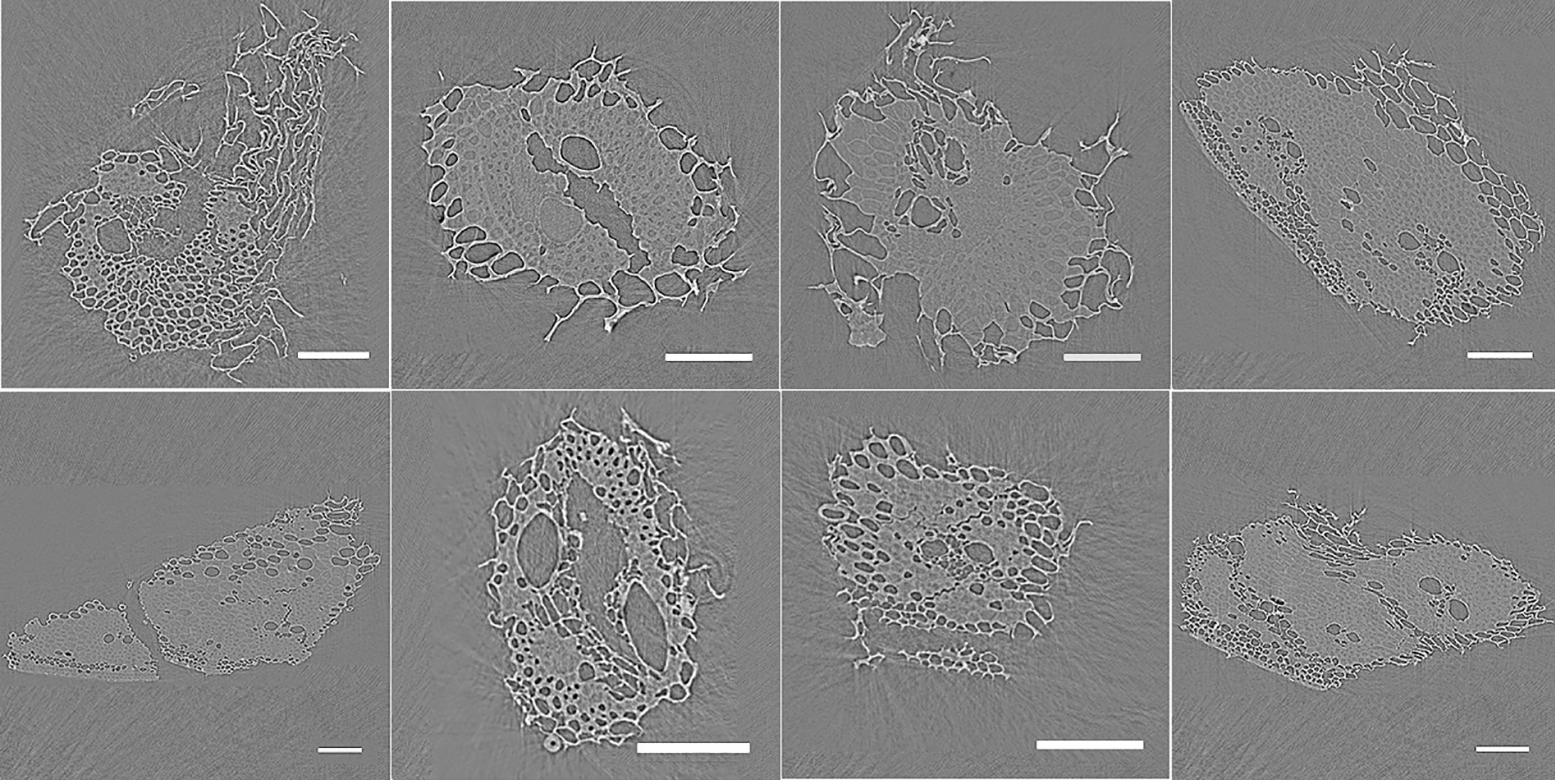
Selected cross section images of eight particles of fresh sugarcane bagasse. The image contrast allows discriminating air (darker), water, and cell walls (lighter). Scale bars: 150 μm.

The cross-sections images of bagasse particles show cells filled with air side by side with cells filled with water (Fig 3). This result proves our hypothesis that fresh bagasse has part of the cells filled with water. In addition, we observe patterns of preferential hydration depending on cell type. More than 90% of xylem vessels are dry, and so do most parenchyma cells. On the other hand, the fibrous cells surrounding the xylem vessels are in most cases full of water. The epidermis region has intermediate behavior, showing both dry and wet cells, and it is not possible to discriminate a preferred hydration state. Noteworthy, the larger volumes (xylem vessels and parenchyma cells) are preferentially dry, which would be consistent with their weaker capillary forces (because of larger sizes) being insufficient to hold the water. An alternative and complementary explanation for their dryness is the connectivity with the external particle environments: xylem vessels are natural conduits for water, while parenchyma cells are in most cases ruptured with the purpose of releasing the cane juice.

**Fig 4:**
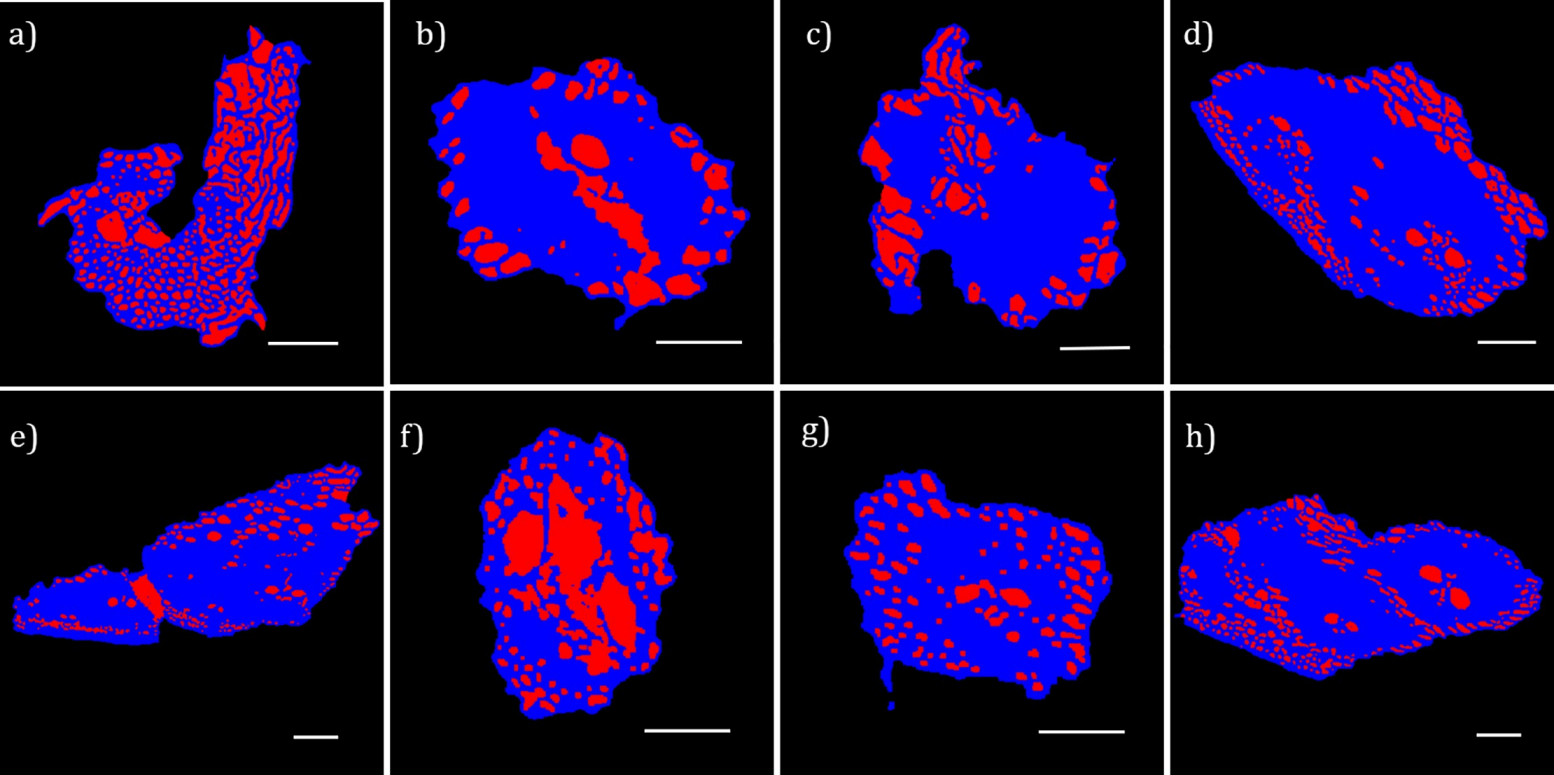
Cross-section images after determination of the particles contours and image segmentation. The exterior of the particle (black) is discriminated from internal air (red) and the cell walls plus internal water (blue). These segmented images allow the estimation of the air volume%. Scale bars: 150 μm.

The air volume percentage in the particles can be estimated from cross-section images such as those in Fig 3. After defining the contour of each particle and segmenting the internal air, %air is simply calculated as the ratio between the red area to the blue+red area (Fig 4). The following air volume percentages were obtained from the cross-sections shown in Fig 4. a: 42%, b: 22%, c: 22%, d: 18%, e: 17%, f: 42%, g: 19%, h: 18%. Unfortunately, water could not be discriminated from cell walls in this image segmentation process due to insufficient contrast. In addition, we were unable to accurately automate the image processing to apply it to the whole set of 3D stacks. Nevertheless, it is noteworthy that the estimated air% is quite variable (17–42%). Moreover, in agreement with having intracellular volumes partly occupied by water, this range of air volume% is consistently below the 62–65% calculated based on the difference between the 1.5 g/cm^3^ cell wall density and the 0.52–0.57 g/cm^3^ mean particle envelope densities previously reported for the fibrous particles of dry sugarcane bagasse [15,16].

**Fig 5:**
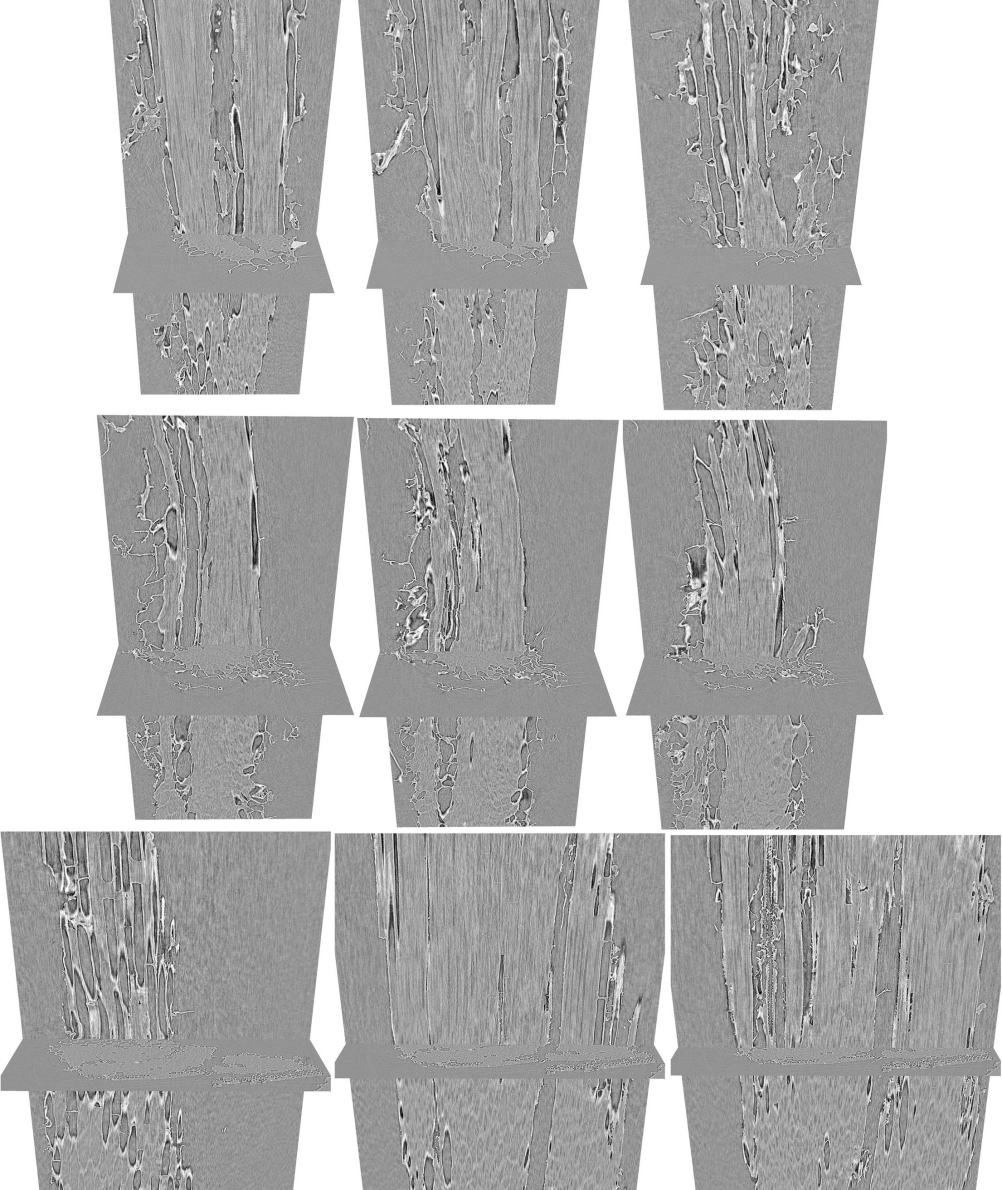
Longitudinal views of three selected bagasse particles. These particles are also shown at the panel of Fig 3. The longitudinal views are orthoslices from progressive depths.

Longitudinal views of the bagasse particles provide additional insight about the cells with preferential retention of water (Fig 5). The observed cellular volumes are filled with either water or air, without notable cases of partial filling with both water and air in the same cell. Moreover, the filled cells are in most cases fibers with sclerified walls that appear to have preserved their integrity despite the mechanical actions of sugarcane crushing. Hence, most water volumes seem to be held by integer cells with preserved cell walls.

Based on this result, it is interesting to discuss the water hold up in terms of two possible mechanisms: preservation *versus* reabsorption. By preservation, we mean the observed water is preserved from the native sugarcane stalks, being unaltered by the crushing. By reabsorption, we mean the native water is expelled by compression between the roller mills but is partly reabsorbed when the pressure is released. Sugar extraction in roller mills is modelled by the reabsorption phenomenon [4], but most sugar comes from the juicy parenchyma, which is mostly disintegrated. Therefore, the well stablished reabsorption models in sugarcane technology do not rule out that a relatively small fraction of the water could be partly preserved from the native stalk. We speculate this is the case of the water found in sclerified fiber cells with preserved integrity. If this is the case, the observed water is indeed juice containing additional sugar, presumably the 2–4% unextractable by conventional means. We speculate tailored shredding or cutting, perhaps cutting along fiber length, could be a viable industrial strategy to liberate this additional juice from fiber cells.

### Analysis of mineral particles

**Fig 6:**
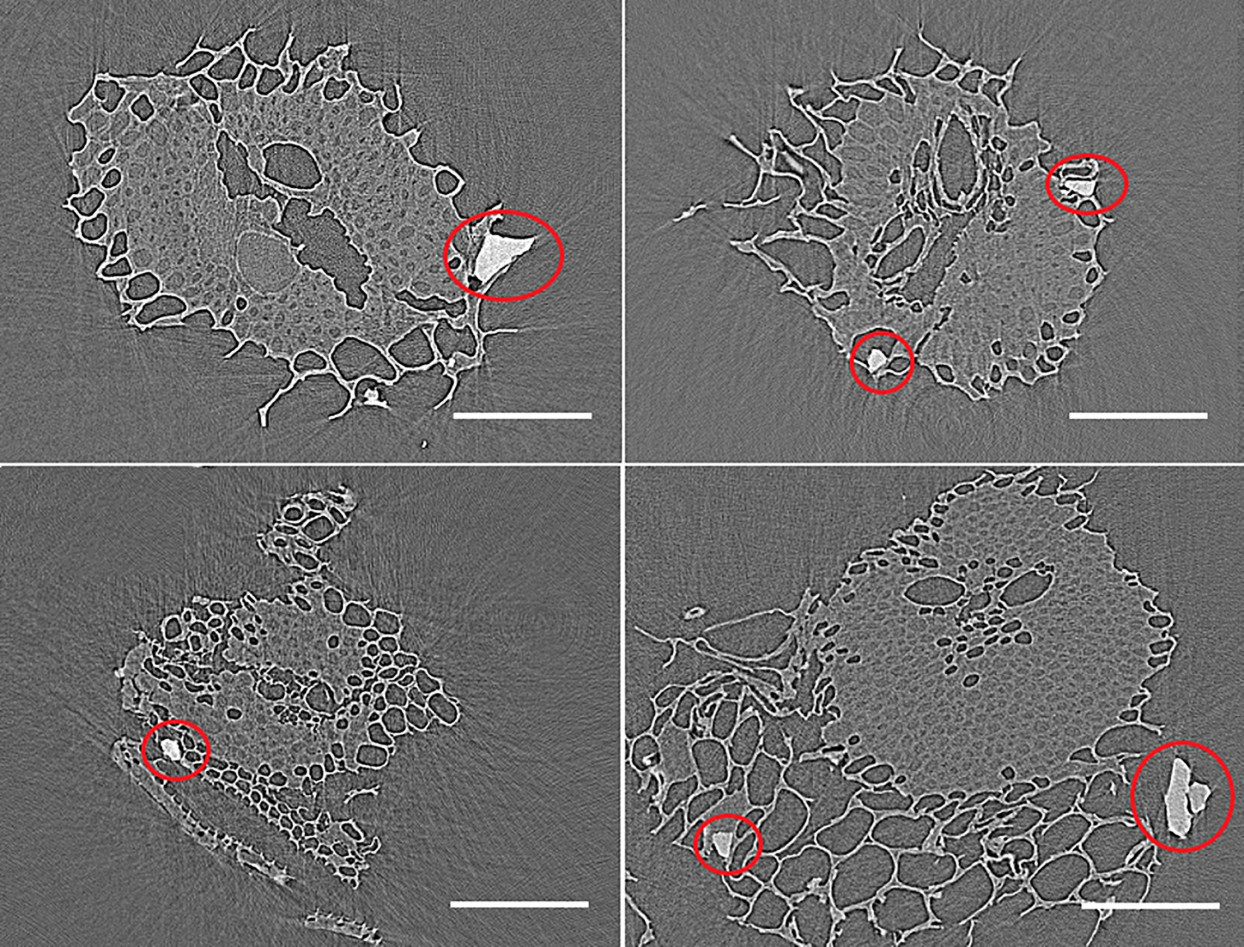
Visualization of mineral particle. Selected cross-section images showing mineral particles (red circles) adhered to wet bagasse. Scale bars: 150 μm.

**Fig 7:**
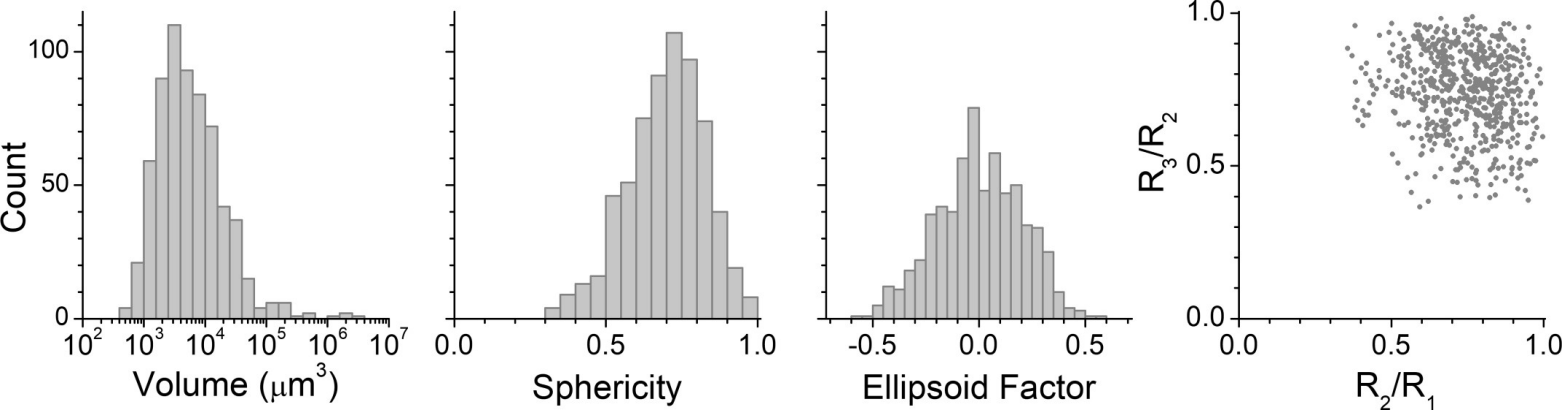
Characteristics of mineral particles observed in wet bagasse. Distributions of volume, sphericity and ellipsoid factor, and scatter plot in Flinn diagram (R_3_/R_2_ vs. R_2_/R_1_).

Mineral particles are observed as high contrast bodies in the microtomograms (Fig 6). Their presence in bagasse is primarily attributed to soil particles that come to the mill as sugarcane impurity and end up adhered and impregnated in the bagasse structure [8]. The image processing workflow identified and analyzed a total of 650 mineral particles in the 23 microtomograms. Distributions of volume, sphericity, and ellipsoid factor are shown in Fig 7, together with the Flinn diagram. It is worth comparing these metrics with the previous study that used an identical particle morphometry method, but analyzed a different bagasse in dry state [8]. Here (Fig 7) as well as in the previous study [8], ellipsoid factors are distributed around zero and data points are scattered close to the upper right corner of the Flinn diagram (Fig 7). This indicates a tendency for spherical particle shapes, without preference for disk- or rod-like morphologies. Sphericity values are mostly between 0.5 and 1.0 (Fig 7), which although lower than in the previous study [8], still supports a tendency for rounded shapes.

As for the particle sizes, mineral particles in this study are larger, with volumes ranging from 500 to 3×10^6^ μm^3^ (Fig 7) compared to a range between 50 and 2×10^4^ μm^3^ observed previously [8]. Calculating the cubic root (volume^1/3^) to have a length dimension, particle sizes range from 8–140 μm in the present study compared to 4-27 μm in the previous one. Since the bagasse samples were different, differences in the mineral particles are not surprising. Nevertheless, two factors may help explaining the different size ranges. First, the pixel binning of this work degrades spatial resolution, thus increasing the size of the smallest detectable particle. Second, in the wet bagasse of this work, water promotes adhesion between mineral particles and bagasse, helping to keep larger mineral particles attached to the biomass. Finally, the mineral particles of this work are in most cases close to or adhered to bagasse external surfaces (Fig 6), which reflect their size being too big to penetrate the tissues.

## Conclusion

Fresh bagasse was collected in a sugarcane mill and immediately transported to a synchrotron X-ray microtomography beamline, where selected particles were imaged. Non-invasive 3D images with micrometric resolution were acquired, showing water volumes located inside bagasse. The fibrous cells surrounding the xylem vessels were often found full of water. We speculated part of the observed water is indeed juice preserved from the native stalk, held up by sclerified fiber cells that preserved their integrity during sugarcane crushing. On the other hand, most of xylem vessels and parenchyma cells were dry (*i*.*e*., filled with air). The epidermis region showed intermediate behavior, showing both dry and wet cells in similar proportions. Particles of mineral impurities with sizes 8–140 μm were also observed. They were in most cases adhered to the external surfaces of the bagasse particles, with water presumably contributing to the adhesion. These results provided novel insights about water-related phenomena taking place during sugarcane crushing and indicated new avenues for advancing sugarcane processing technology and bagasse valorization.
